# Activation of the dopamine 1 and dopamine 5 receptors increase skeletal muscle mass and force production under non-atrophying and atrophying conditions

**DOI:** 10.1186/1471-2474-12-27

**Published:** 2011-01-26

**Authors:** Deborah L Reichart, Richard T Hinkle, Frank R Lefever, Elizabeth T Dolan, Jeffrey A Dietrich, David R Sibley, Robert J Isfort

**Affiliations:** 1 Research Division, Procter & Gamble, Mason, OH, USA; 2 Molecular Neuropharmacology Section, NINDS, NIH, Bethesda, MD, USA

## Abstract

**Background:**

Control of skeletal muscle mass and force production is a complex physiological process involving numerous regulatory systems. Agents that increase skeletal muscle cAMP levels have been shown to modulate skeletal muscle mass and force production. The dopamine 1 receptor and its closely related homolog, the dopamine 5 receptor, are G-protein coupled receptors that are expressed in skeletal muscle and increase cAMP levels when activated. Thus we hypothesize that activation of the dopamine 1 and/or 5 receptor will increase skeletal muscle cAMP levels thereby modulating skeletal muscle mass and force production.

**Methods:**

We treated isolated mouse tibialis anterior (TA) and medial gastrocnemius (MG) muscles in tissue bath with the selective dopamine 1 receptor and dopamine 5 receptor agonist SKF 81297 to determine if activation of skeletal muscle dopamine 1 and dopamine 5 receptors will increase cAMP. We dosed wild-type mice, dopamine 1 receptor knockout mice and dopamine 5 receptor knockout mice undergoing casting-induced disuse atrophy with SKF 81297 to determine if activation of the dopamine 1 and dopamine 5 receptors results in hypertrophy of non-atrophying skeletal muscle and preservation of atrophying skeletal muscle mass and force production.

**Results:**

In tissue bath, isolated mouse TA and MG muscles responded to SKF 81297 treatment with increased cAMP levels. Treating wild-type mice with SKF 81297 reduced casting-induced TA and MG muscle mass loss in addition to increasing the mass of non-atrophying TA and MG muscles. In dopamine 1 receptor knockout mice, extensor digitorum longus (EDL) and soleus muscle mass and force was not preserved during casting with SKF 81297 treatment, in contrast to significant preservation of casted wild-type mouse EDL and soleus mass and EDL force with SKF 81297 treatment. Dosing dopamine 5 receptor knockout mice with SKF 81297 did not significantly preserve EDL and soleus muscle mass and force although wild-type mouse EDL mass and force was significantly preserved SKF 81297 treatment.

**Conclusions:**

These data demonstrate for the first time that treatment with a dopamine 1/5 receptor agonist results in (1) significant preservation of EDL, TA, MG and soleus muscle mass and EDL muscle force production during periods of atrophy and (2) hypertrophy of TA and MG muscle. These effects appear to be mainly mediated by both the dopamine 1 and dopamine 5 receptors.

## Background

Dopamine has multiple central and peripheral physiological effects. In the brain, dopamine controls a multitude of functions including locomotor activity, cognition, emotion, positive reinforcement, food intake and endocrine regulation. In the periphery, dopamine modulates cardiovascular activity (both cardiac and vascular function), catecholamine release, hormone secretion, renal function and gastrointestinal motility [1]. Dopamine mediates its action via the 5 known dopamine receptors (D1-D5). These five receptors can be subdivided into two general groups, the D1 receptor/D5 receptor group (D1-like) and the D2 receptor/D3 receptor/D4 receptor group (D2-like), based on their molecular structures, pharmacological activities, and physiological functions [1-5]. The D1-like receptors predominantly signal by coupling to Gαs which leads to the activation of adenylyl cyclase and the formation of cAMP [1,3,6,7]. The D2-like receptors signal mainly by coupling to Gαi, thereby inhibiting the activity of adenylyl cyclase [1,3,8,9]. In addition, alternative G protein coupling has been described for both the D1-like receptor and the D2-like receptor groups under specific conditions [10-13]. The dopamine receptors have been cloned from many species, including humans, with splice variants of many of the dopamine receptors identified [1,3]. Gene expression analysis of the dopamine receptors has demonstrated that the D1-like receptor group is expressed centrally in many areas of the brain and peripherally in blood vessels, the adrenal gland, skeletal muscle and the kidneys [1,4,14-17]. The D2-like receptor group is also expressed centrally in many areas of the brain and peripherally in the pituitary, blood vessels, the heart, the adrenal gland, and the kidneys [1,4,15,17]. Pharmacologically, agonists and antagonists that functionally differentiate dopamine receptors have been described and have been useful in matching biological activity with individual dopamine receptors [1,17,18].

Skeletal muscle is a plastic tissue which readily changes mass in response to alterations in physiological demand for work and metabolic need. Loss of skeletal muscle mass (atrophy) can be initiated by a variety of stimuli including disuse, nerve damage, glucocorticoid use, sepsis, cachexia, chronic pulmonary obstructive disease, congestive heart failure and muscular dystrophy [19-28]. Several agents have been shown to modulate skeletal muscle mass including anabolic steroids, growth hormone, insulin-like growth factor I, corticotrophin releasing factor 2 receptor agonists, phosphodiesterase 4 inhibitors, vasoactive intestinal peptide 2 receptor agonists and beta 2 adrenergic receptor agonists [29-36]. We have previously demonstrated that modulation of skeletal muscle cAMP levels by activation of receptors that are positively coupled to adenylate cyclase and by inhibition of phosphodiesterases that degrade cAMP increase skeletal muscle mass under both physiological and pathological conditions [31-34]. We were therefore interested in determining if activation of the dopamine 1 and dopamine 5 receptors would increase skeletal muscle cAMP thereby resulting in increased muscle mass and force. To do this, we utilized a selective dopamine 1/dopamine 5 receptor agonist (SKF 81297) to treat wild-type, dopamine 1 receptor knockout and dopamine 5 receptor knockout mice and measured skeletal muscle cAMP levels, skeletal muscle mass and skeletal muscle force production.

## Methods

SKF81297 and theophylline were purchased from Sigma Chemical Company (St. Louis, MO). Male C57BL6 mice weighing approximately 20-30 grams were purchased from Charles River (Kingston, NY). The dopamine 1 receptor knockout mice and their corresponding wild-type mice were purchased from Jackson Laboratories (Bar Harbor, ME). The dopamine 5 receptor knockout mice and their corresponding wild-type mice were obtained from Dr. David Sibley (NIH, Bethesda, MD) and bred at Procter & Gamble. Mice were single-housed and acclimatized to the conditions of the facility for approximately 1 week before use. Mice had access to lab chow and water ad libitum and were subjected to standard conditions of humidity, temperature and a 12 hour light cycle. All animal studies described in this report were conducted in compliance with the US Animal Welfare Act, the rules and regulations of the State of Ohio Departments of Health, Procter & Gamble's policy on research involving animals with strict oversight for care/welfare and were approved by the Procter & Gamble Institutional Animal Care and Use Committee.

### Evaluation of cAMP generation by dopamine 1/5 receptor agonist treatment of skeletal muscle in tissue bath

Evaluation of muscle cAMP levels was performed as described by us previously [32,33]. Briefly, skeletal muscle tissue baths were prepared by dissecting the medial gastrocnemius and tibialis anterior muscles from both the right and left legs of mice and pinning the muscles at resting length on rubber supports adhered to glass microscope slides. The muscles were then incubated with continuous aeration with 95% oxygen/5% carbon dioxide in 25 ml of a 30°C pH 7.4 Krebs-Ringers solution. After 15 minutes this solution was changed and was replaced with 25 ml of pH 7.4 Krebs-Ringer solution containing 25 mM theophylline and the muscles were incubated at 30°C with continuous aeration for an additional 15 minutes. 25 mM theophylline was added to the incubation bath in order to inhibit phosphodiesterase mediated breakdown of cAMP. The test compounds were added to the pH 7.4 Krebs-Ringer solution containing 25 mM theophylline and the muscles were incubated for 1 hour after which they were removed from the baths, blotted dry and snap frozen in liquid nitrogen. The muscles were prepared for cAMP analysis by grinding into a fine powder in liquid nitrogen using a mortar and pestle followed by dissolving the powdered muscle in lysis buffer and incubating for 30 minutes as specified by the cAMP kit manufacturer (Amersham Pharmacia Biotech Inc., Piscataway, NJ). cAMP levels were then measured using the Amersham Pharmacia Biotech cAMP measurement kit RPN225 essentially as recommended by the manufacturer. Each experiment was performed in triplicate. The data is presented as fold increase over control with 1 fold being equivalent to background rate.

### Leg casting disuse-induced muscle atrophy model

Lower leg casting disuse-induced muscle atrophy was performed as described previously [32,33]. Briefly, mice were anesthetized with isoflurane and the lower right leg was casted from the knee to the toes with heat activated casting material (Vet Lite, Kruuse Inc., Marslev, Denmark). The test materials (vehicle or 30 mg/kg SKF 81297) were administered by once daily subcutaneous injections in the midscapular region. Fourteen days after casting, animals were euthanized by carbon dioxide asphyxiation followed by cervical dislocation. The cast was removed and the lower leg muscles (tibialis anterior, medial gastrocnemius, extensor digitorum longus and soleus) were dissected rapidly, cleaned of tendons/connective tissue and weighed.

### Muscle functional analysis

Ten days after application of casts, eight mice per treatment group were anesthetized using isoflurane. The casts were removed and the right legs shaved and the extensor digitorum longus and soleus muscles exposed. Silk sutures (5-0) were tied to the proximal and distal tendons of the EDL and soleus muscles and the muscles were removed. The muscles were then immediately placed into a bath with oxygenated Ringer's solution (95% O_2 _and 5% CO_2_), tied to a force transducer and a fixed post within the bath and stimulated with single and trains of electrical pulses (1 Hz and 10-200 Hz, respectively) in order to assess the muscles ability to generate force. Upon completion of the force generation analysis, the muscles were removed from the baths, blotted dry and weighed.

### Statistical analysis

Statistical analysis of the data was performed using an ANCOVA model with treatment effect and starting weight as the covariates. Pairwise comparisons for all end-points were generated using least-square means (SAS, Cary, North Carolina), adjusted for unequal sample sizes and starting weight.

## Results

### cAMP generation following dopamine 1 and dopamine 5 receptor activation in skeletal muscle

In order to evaluate if activation of the dopamine 1 and dopamine 5 receptors would increase cAMP levels in skeletal muscle, mouse TA and MG muscles were removed, incubated in tissue baths, treated with the dopamine 1 and dopamine 5 receptor selective agonist SKF 81297 and cAMP levels measured. As can be seen in Table 1, treatment of TA muscle with SKF 81297 (in the presence of theophylline which was added to inhibit phosphodiesterases and prevent the breakdown of cAMP during the collection period) resulted in a statistically significant (p < 0.05) 1.5 fold increase in cAMP levels over theophylline alone, while treatment of the MG muscle with SKF 81297 plus theophylline resulted in a statistically significant 1.9 fold increase in cAMP levels over theophylline alone. Thus, treatment of skeletal muscle with a dopamine 1 and dopamine 5 receptor selective agonist increases muscle cAMP levels.

**Table 1:** Effect of dopamine 1/5 receptor agonist treatment on skeletal muscle cAMP levels.

Treatment	Tibialis Anterior cAMP levels +/- SEM (femptomoles/mg)	Medial Gastrocnemius cAMP levels +/- SEM (femptomoles/mg)
Theophylline	329.6 +/- 62.6	286.4 +/-58.8

SKF 81297 + theophylline	486.6 +/- 81.4*	535.3 +/-53.6*

* - statistically significant (p < 0.05) versus theophylline.

### Treatment of mice undergoing disuse induced atrophy with SKF 81297

SKF 81297 was administered to C57Bl6 mice with their lower right leg casted (knee to ankle walking cast) in order to evaluate the effect of activation of the dopamine 1 and dopamine 5 receptors on atrophying (right lower leg muscle) and non-atrophying (left lower leg muscle) skeletal muscle mass. As can be seen in Table 2, once daily treatment of casted mice with 30 mg/kg SKF 81297 for fourteen days resulted in statistically significant (p < 0.05) increase in the mass of the uncasted left leg TA (+9%) and MG (+7%) muscles when compared to vehicle control. In addition, treatment with SKF 81297 resulted in a statistically significant (p < 0.05) reduction in the casted right leg TA (32%) and MG (49%) atrophy-induced muscle mass loss compared to vehicle control.

**Table 2:** Effect of dopamine 1/5 receptor agonist SKF 81297 on atrophying (right, casted) and non-atrophying (left, uncasted) C57Bl6 mouse tibialis anterior and medial gastrocnemius muscle mass.

Parameter	Treatment	Number of mice	Mean	Standard Error
Initial body mass (grams)	Vehicle 1	8	23.4	0.2
	SKF 81297	8	23.9	0.2

Final body mass (grams)	Vehicle 1	8	23.5	0.2
	SKF 81297	8	24.2	0.3

Right Casted Tibialis Anterior (milligrams)	Vehicle 1	8	30.9	1.0
	SKF 81297	8	33.3*	0.7

Right Casted Medial Gastrocnemius (milligrams)	Vehicle 1	8	32.4	1.1
	SKF 81297	8	36.3*	0.8

Left Uncasted Tibialis Anterior (milligrams)	Vehicle 1	8	38.3	0.7
	SKF 81297	8	41.9*	0.7

Left Uncasted Medial Gastrocnemius (milligrams)	Vehicle 1	8	40.4	0.7
	SKF 81297	8	43.5*	0.5

* - statistically significant (p < 0.05) versus appropriate vehicle control.

### Evaluation of the effect of SKF 81297 treatment on skeletal muscle mass and force production in wild-type, dopamine 1 receptor knockout and dopamine 5 receptor knockout mice undergoing disuse induced atrophy

In order to determine (1) which dopamine receptor is responsible for the changes in muscle mass observed following SKF 81297 treatment and (2) if both mass and force production are preserved, we evaluated dopamine 1 receptor and dopamine 5 receptor knockout mice, along with their genetically matched wild-type control animals, in the mouse casting-induced atrophy model. Casting resulted in a statistically significant (p < 0.05) loss of both wild-type mouse EDL muscle mass and force (-24% mass/-22% force) and dopamine 1 receptor knockout mouse EDL muscle mass and force (-22% mass/-14% force). Casting also resulted in a loss of wild-type mouse soleus muscle mass and force (-34% mass/-29% force) and dopamine 1 receptor knockout mouse soleus muscle mass and force (-33% mass/-39% force). (Table 3). Dosing with SKF 81297 resulted in a statistically significant (p < 0.05) reduction in the casting-induced loss of wild-type mouse EDL muscle mass and force (+42% mass/+51% force increase compared to vehicle treated animals) and soleus muscle mass and force (+23% mass/+25% force increase compared to vehicle treated animals). In contrast, SKF 81297 treatment did not result in a statistically significant reduction in the casting-induced loss of muscle mass and force in dopamine 1 receptor knockout mouse EDL muscle mass and force (-5% mass/-19% force decrease compared to vehicle treated animals) and soleus muscle mass and force (+9% mass/+2% force increase compared to vehicle treated animals) (Table 3). There was a small increase in casted and SKF 81297 treated wild type mouse body mass compared to uncasted and casted vehicle treated wild type mice. In addition casted SKF 81297 and vehicle treated dopamine 1 receptor knockout mice demonstrated a small decrease in body mass compared to uncasted vehicle treated dopamine 1 receptor knockout mice (Table 3).

**Table 3:** Effects of once daily 30 mg/kg treatment with the dopamine 1/5 receptor agonist SKF 81297 on atrophying skeletal muscle mass in wild-type (+/+ D1R) and dopamine 1 receptor homozygous knockout (-/- D1R) mice

Parameter	Uncasted Vehicle +/+ D1R	Casted Vehicle +/+ D1R	Casted SKF 81297 +/+ D1R	Uncasted Vehicle -/- D1R	Casted Vehicle -/- D1R	Casted SKF 81297 -/- D1R
# mice	12	11	10	11	11	11

Initial body mass (grams)	24.6 (1.0)	24.4 (0.9)	24.1 (1.1)	22.1 (1.1)	22.3 (1.1)	21.6 (0.8)

Final body mass (grams)	24.9 (1.0)	24.2 (0.8)	25.8#* (1.2)	23.6 (1.1)	22.6# (1.1)	23.0# (0.9)

EDL mass (milligrams)	9.8 (0.4)	7.4# (0.4)	8.4#* (0.5)	9.6 (0.4)	7.5# (0.5)	7.4# (0.4)

EDL Po (milliNewtons)	402.8 (16.0)	315.0# (15.6)	359.5#* (22.7)	371.0 (11.0)	317.8# (22.1)	307.6# (16.3)

Soleus mass (milligrams)	7.6 (0.3)	5.0# (0.3)	5.6#* (0.4)	6.9 (0.3)	4.6# (0.3)	4.8# (0.3)

Soleus Po (milliNewtons)	209.3 (6.1)	149.0# (6.8)	164.2# (12.0)	196.2 (11.3)	119.7# (7.1)	121.4# (7.0)

All data given as mean + (SEM). * - statistically significant (p < 0.05) versus casted vehicle; # - statistically significant (p < 0.05) versus uncasted vehicle.

Evaluation of dopamine 5 receptor knockout mice revealed that casting resulted in a statistically significant (p < 0.05) muscle mass and force loss in both wild-type mouse EDL muscle (-32% mass/-32% force) and dopamine 5 receptor knockout mouse EDL muscle (-33% mass/-33% force). Casting resulted in a similar response in wild-type mouse soleus muscle (-40% mass/-40% force) and dopamine 5 receptor knockout mouse soleus muscle (-32% mass/-35% force) (Table 4). Treatment with SKF 81297 resulted in a reduction in the casting-induced loss of wild-type mouse EDL muscle mass and force (significant +21% mass/+28% force increase compared to vehicle treated animals) with no significant preservation of casting-induced loss of wild-type mouse soleus muscle mass and force (+3% mass/+11% force increase compared to vehicle treated animals). Treatment with SKF 81297 resulted in a small and statistically non-significant preservation of dopamine 5 receptor knockout mouse EDL muscle mass and force (non-significant +10% mass/+20% force increase compared to vehicle treated animals) with no preservation of dopamine 5 receptor knockout mouse soleus muscle mass and force (non-significant -4% mass/-4% force increase compared to vehicle treated animals) (Table 4).

**Table 4:** Effects of daily 30 mg/kg dosing with the dopamine 1/5 receptor agonist SKF 81297 on atrophying skeletal muscle mass and force in wild-type (+/+ D5R) and dopamine 5 receptor homozygous knockout (-/- D5R) mice.

Parameter	Uncasted Vehicle +/+ D5R	Casted Vehicle +/+ D5R	Casted SKF 81297 +/+ D5R	Uncasted Vehicle -/- D5R	Casted Vehicle -/- D5R	Casted SKF 81297 -/- D5R
# mice	12	12	12	12	12	12

Initial body mass (grams)	23.0 (0.5)	22.7 (0.3)	22.3 (0.6)	22.3 (0.3)	22.5 (0.4)	21.8 (0.4)

Final body mass (grams)	23.0 (0.4)	21.6# (0.4)	22.8* (0.7)	23.1 (0.4)	21.3# (0.5)	22.4#* (0.4)

EDL mass (milligrams)	8.8 (0.2)	6.0# (0.2)	6.6#* (0.3)	8.8 (0.2)	5.9# (0.2)	6.2# (0.2)

EDL Po (milliNewtons)	351.3 (10.8)	237.3# (8.0)	269.7#* (13.6)	348.4 (10.5)	233.1# (9.2)	256.0# (12.1)

Soleus mass (milligrams)	7.5 (0.2)	4.5# (0.2)	4.6# (0.2)	7.4 (0.2)	5.0# (0.2)	4.9# (0.2)

Soleus Po (milliNewtons)	202.2 (8.2)	120.7# (8.1)	130.0# (8.5)	204.2 (7.6)	132.8# (8.4)	129.9# (7.1)

All data given as mean + (SEM). * - statistically significant (p < 0.05) versus casted vehicle; # - statistically significant (p < 0.05) versus uncasted vehicle.

There was a small increase in casted and SKF 81297 treated wild type mouse body mass compared to casted vehicle treated wild type mice with casted vehicle treated wild type mice having a small decrease in body mass compared to uncasted vehicle treated wild type mice. In addition, casted SKF 81297 and vehicle treated dopamine 5 receptor knockout mice demonstrated a small decrease in body mass compared to uncasted vehicle treated dopamine 1 receptor knockout mice with casted SKF 81297 dopamine 5 receptor knockout mice having a small increase in body mass compared to vehicle treated dopamine 5 receptor knockout mice (Table 4).

## Discussion

In this report we demonstrate for the first time that activation of the dopamine 1/5 receptors results in increased skeletal muscle cAMP, increased non-atrophying muscle mass and reduced atrophy-induced loss of muscle mass and force production. By using knockout mice to differentiate the effects of activation of the dopamine 1 receptor from that of the dopamine 5 receptor, we demonstrate that both the dopamine 1 and dopamine 5 receptors mediate the anti-atrophy effects of the dopamine 1/5 receptor selective agonist SKF 81297. Genetic removal of the dopamine 1 receptor (with maintenance of the dopamine 5 receptor) results in a complete loss of the SKF 81297 mediated EDL mass/force preservation, data consistent with the idea that the dopamine 1 receptor mediates the effects of SKF 81297. In contrast, genetic removal of the dopamine 5 receptor (with maintenance of the dopamine 1 receptor) resulted in a partial loss of SKF 81297 mediated EDL mass/force preservation, data that is inconsistent with the prior observation that the dopamine 1 receptor mediates the effects of SKF 81297. Together these findings indicate that both the dopamine 1 and dopamine 5 receptors mediate the anti-atrophy effects of SKF 81297. One potential mechanism to explain the above observations is that the dopamine 1 and 5 receptors dimerize such that the dopamine 1/dopamine 5 receptor heterodimers are the maximally effective combination while dopamine 1 receptor homodimers are less effective and dopamine 5 receptor homodimers are ineffective. Heterodimerization and homodimerization of both the dopamine 1 and dopamine 5 receptors have been described with heterodimers displaying behaviors not observed with homodimers in response to SKF 81297 stimulation [37,38]. Additional work will be required to confirm this potential mechanism and clearly understand the effect of potential dopamine 1/5 receptor homodimers and heterodimers have on receptor signaling in myocytes.

In general, the casting-induced changes in muscle mass and force were of a similar magnitude in wild-type and dopamine receptor knockout mice, although several exceptions were noted including: (1) a larger change in casting-induced EDL mass loss (-22%) compared to casting-induced EDL force loss (-14%) in dopamine 1 receptor knockout mice; (2) a larger change in casting-induced EDL force loss (-19%) compared to casting-induced EDL mass loss (-5%) in SKF 81297 treated dopamine 1 receptor knockout mice; and (3) a larger preservation of EDL muscle force (+20%) compared to EDL muscle mass (+10%) in SKF 81297 treated dopamine 5 receptor knockout mice. Interestingly, no relative difference in EDL muscle mass or force were observed in wild-type mice under any experimental condition and no relative difference in soleus muscle mass or force were observed in wild-type, dopamine 1 receptor knockout mice and dopamine 5 receptor knockout mice under any experimental conditions. At present it is unclear as to the mechanism that leads to the difference in EDL muscle force versus mass changes in the dopamine receptor knockout mice, although in another muscle cAMP modulating receptor system, it has been reported that short term activation of the beta 2 adrenergic receptor increases EDL muscle force without increasing muscle mass in wild-type mice [39]. The greater reductions in EDL force relative to mass that we observed in dopamine receptor knockout mice may indicate that the dopamine 1 and dopamine 5 receptors function to modulate skeletal muscle force in a manner that is dissociated from the effect these receptors have on modulating skeletal muscle mass (similar to that observed with the beta 2 adrenergic receptor), although the effects of the dopamine 1 and dopamine 5 receptors on muscle force generation appear to be more complicated than that of the beta 2 adrenergic receptor mediated effects since we did not observe an increase in EDL force versus mass in wild-type mice treated with SKF 81297 (both increased similarly). Further study will be required to understand the role of the dopamine 1 and dopamine 5 receptors in mediating EDL muscle force.

All of the four lower leg muscles examined (MG, TA, EDL and soleus) responded to SKF 81297 treatment with a reduction in atrophy-induced mass loss, although there was some experiment-to-experiment variability in the magnitude of the response. In the experiment in which we examined the effects of SKF 81297 treatment on both casted and non-casted TA and MG muscles, the magnitude of the reduction in muscle mass loss in the casted leg muscle (3.2 mg averaged difference in muscle mass between SKF 81297 and vehicle treated muscles) and the gain in muscle mass in the uncasted leg muscle (3.4 mg averaged difference in muscle mass between SKF 81297 and vehicle treated muscles) was similar. The fact that the absolute gain in muscle mass was similar in both the SKF 81297 treated casted and uncasted leg muscles indicates that there was minimal casting induced hypertrophy of the uncasted leg, with the increase in muscle mass coming from the effect of SKF 81297 treatment. This is consistent with our previous observations using the lower leg casting method, in which we did not observe differences in uncasted lower leg muscle mass when we compared casted and uncasted animals. The lack of casting induced hypertrophy of the uncasted leg results from the fact that the lower leg casts used are relatively small and do not impede the movement of the mice since they are walking casts (RJI personal observation). The observation that different muscles, in different mice strains and in different physiological states (normal versus atrophying) respond similarly to SKF 81297 demonstrates a robustness in the dopamine 1/5 receptor response.

Previous reports have demonstrated several major roles for dopamine receptors in skeletal muscle. The first involves modulation of glycogen concentration, glycogen synthase activity and insulin-stimulated glucose transport in skeletal muscle [40]. These effects are mediated by elevated muscle cAMP levels and are the result of a direct effect of levodopa action on skeletal muscle since the effects are observed in isolated muscle in tissue bath [40]. A second reported effect of dopamine on skeletal muscle is an improvement in diaphragm muscle force development and a reduction in diaphragm apoptosis in vitro [41]. Finally, dopamine has been reported to reduce diaphragm fatigue in vivo by increasing diaphragm muscle blood flow [42]. Together these results demonstrate that dopamine has multiple roles in skeletal muscle including modulating energy storage, modulating muscle force production and controlling muscle fatigue. While the observation that dopamine 1/5 receptors are expressed in skeletal muscle suggest that these effects may be mediated by the dopamine 1/5 receptors, the use of the non-selective dopamine receptor agonists (dopamine, levodopa and l-dopa) makes these conclusions somewhat tentative for the following reasons. First, it has been demonstrated that in skeletal muscle cells, dopamine (a metabolite of levodopa) can activate the beta 2 adrenergic receptor and the effects of dopamine can be blocked with a beta 2 adrenergic receptor antagonist but not a dopamine receptor antagonist or an alpha adrenergic receptor antagonist [43,44]. Thus, the effects of dopamine on skeletal muscle that have been previously described may actually be the result of activation of the beta 2 adrenergic receptor and not the result of direct dopamine receptor activation. In support of this, Smith et al. [40] demonstrate that the effects of levodopa on glycogen storage could be reversed by using a beta 2 adrenergic receptor antagonist. In addition, Pierce et al. [41] argue that the effects of dopamine on muscle performance may be mediated via the beta 2 adrenergic receptor. In contrast to these previous studies, our studies were performed with a very selective dopamine 1/dopamine 5 receptor agonist that is devoid of beta 2 adrenergic receptor affinity [45]. In addition, we demonstrate that the increase in muscle mass and force production observed with the selective dopamine 1/dopamine 5 receptor agonist could be abrogated when the dopamine 1 receptor and to a lesser extent the dopamine 5 receptors are genetically deleted. Together, our data argues that the skeletal muscle effects we observed including the increase in mass and force production are the result of direct stimulation of the dopamine 1 and the dopamine 5 receptors and do not result from off-target activation of the beta 2 adrenergic receptor. Thus, this report demonstrates for the first time that activation of the dopamine 1 and dopamine 5 receptors results in increased cAMP production and increased muscle mass and force production. Additional studies using selective dopamine receptor agonists and mice lacking specific dopamine receptors will be required before we understand if the effects of dopamine on skeletal muscle glycogen utilization, insulin resistance, fatigue and force output are mediated by the dopamine 1 and the dopamine 5 receptors.

## Conclusion

We demonstrate for the first time that activation of the dopamine 1 receptor and to a lesser extend the dopamine 5 receptor results in modulation of skeletal muscle mass and force under both atrophying and non-atrophying conditions.

## Competing interests

This work was supported by the Procter & Gamble Company. DLR, RTH, FRL, ETD, JAD and RJI are, or were at the time these studies were performed, employees of the Procter & Gamble Company.

## Authors' contributions

All authors made substantial intellectual, design and/or executional contributions to this study as follows: DLR provided executional contributions; RTH provided intellectual, design and executional contributions; FRL provided executional contributions; ETD provided executional contributions; JAD provided executional contributions; DRS provided executional contributions; and RJI provided intellectual, design and executional contributions. All authors gave final approval of the manuscript

## Pre-publication history

The pre-publication history for this paper can be accessed here:

http://www.biomedcentral.com/1471-2474/12/27/prepub
